# Combined Integrative RNA-Seq and Serological sIgE Analysis Enhances Understanding of Fish Allergen Profiles and Diagnostic Strategy for Fish Allergy

**DOI:** 10.3390/ijms251910784

**Published:** 2024-10-07

**Authors:** Zhong-Yi Liu, Christine Yee Yan Wai, Agnes Sze Yin Leung, Wai Hung Chan, Jaime Sou Rosa Duque, Ivan Cheuk San Lam, James Wesley Cheng, Jason Ka Chun Sit, Noelle Anne Ngai, Po Ki Ho, Gilbert T. Chua, Qun Ui Lee, Oi Man Chan, Yat Sun Yau, Joshua Sung Chi Wong, David Chi Kong Luk, Marco Hok Kung Ho, Mike Yat Wah Kwan, Man Fung Tang, Nicki Yat Hin Leung, Ting Fan Leung

**Affiliations:** 1 Department of Paediatrics, Prince of Wales Hospital, The Chinese University of Hong Kong, Hong Kong SAR, China; zhongyiliu@cuhk.edu.hk (Z.-Y.L.); christineyywai@cuhk.edu.hk (C.Y.Y.W.); agnes.syl@cuhk.edu.hk (A.S.Y.L.); 12.jason25@link.cuhk.edu.hk (J.K.C.S.); oimanchan@cuhk.edu.hk (O.M.C.); mftang@cuhk.edu.hk (M.F.T.);; 2 Hong Kong Hub of Paediatric Excellence, The Chinese University of Hong Kong, Hong Kong SAR, China; 3 Department of Paediatrics, Queen Elizabeth Hospital, Hong Kong SAR, China; 4 Department of Paediatrics and Adolescent Medicine, The University of Hong Kong, Hong Kong SAR, China; jsrduque@hku.hk; 5 Department of Paediatrics and Adolescent Medicine, Princess Margaret Hospital, Hong Kong SAR, Chinakwanyw1@ha.org.hk (M.Y.W.K.); 6 Department of Paediatrics and Adolescent Medicine, Yan Chai Hospital, Hong Kong SAR, China; 7 Department of Paediatrics and Adolescent Medicine, United Christian Hospital, Hong Kong SAR, China; 8 Department of Paediatrics, Prince of Wales Hospital, Hong Kong SAR, China; 9 Department of Paediatrics and Adolescent Medicine, Queen Mary Hospital, University of Hong Kong, Hong Kong SAR, China

**Keywords:** fish allergy, RNA-seq, allergen discovery, parvalbumin, ImmunoCAP sIgE, component-resolved diagnosis, fish allergenicity ladder

## Abstract

Fish allergy is a significant health concern, with diagnosis and management complicated by diverse fish species and allergens. We conducted a comprehensive RNA-seq analysis of eight fish species to identify allergen profiles, integrating ImmunoCAP sIgE data to explore associations with allergen expression and diagnostic performance. Over 30 putative fish allergens were identified, with varying sequence similarities and expression levels, roughly classifying fish into two groups based on parvalbumin (PV) expression. Higher similarities in allergen expression correlated with stronger sIgE data relationships among fish extracts. High PV expression and conserved PV sequences were linked to elevated sIgE measurements, potentially indicating higher allergenicity. For diagnosis, species-specific extract sIgE remained the best indicator of corresponding fish allergy diagnosis, while incorporating multiple sIgE data enhanced performance. In component-resolved diagnosis (CRD), the current panel with PV alone showed comparable performance to fish extract for PV-high fish allergy, while PV-low fish may require the inclusion of more minor allergens for improved CRD accuracy. This RNA-seq allergen analysis helps reveal fish allergen profiles, classify fish groups, and predict allergenicity, potentially improving CRD design and food management in fish allergy.

## 1. Introduction

Fish, with over 30,000 species, are crucial in modern nutrition but are also among the “Big Nine” allergenic foods, which include milk, eggs, crustacean shellfish, tree nuts, peanuts, wheat, soybeans, and sesame [1,2]. The prevalence of fish allergy varies between 0.2% and 7%, influenced by different consumption habits [3,4,5]. Clinically, fish allergy is a major cause of food-induced anaphylaxis, with a lower likelihood of outgrowing compared to milk or egg allergies [6,7]. Due to the significant cross-reactivity of fish allergy, patients are often advised to avoid all fish, potentially leading to nutritional deficiencies.

Whole fish extracts are commonly used in skin prick tests (SPTs) and ImmunoCAP specific IgE (sIgE) assays [8]. Commercial extracts are currently available for over 20 fish species. Multiple fish extracts are often employed to evaluate whether the subject is allergic to multiple fish [9]. However, it is unclear if these tests, based on representative fish species, effectively cover potential cross-reactivity with other untested fish. Better understanding of fish allergenicity differences could improve allergy management and dietary strategies.

Component-resolved diagnosis (CRD) at the molecular or epitope level has gained attention for precision allergy diagnosis [10]. Fish parvalbumins (PVs) are the major fish allergens with reported sensitization rates of around 90% in different patient cohorts [11,12]. Currently, two recombinant PVs from cod and common carp (rGad c 1 and rCyp c 1) are commercially available. However, it remains uncertain whether they can represent other fish PVs or act as suitable alternatives to fish extracts for different fish allergies. The way towards CRD requires comprehensive understanding of fish allergens at the sequence level.

We hypothesized that fish allergen profiling may reveal different fish allergenicity for better food management and provide novel insights for CRD design. Traditional wet-lab methods involve the preparation of whole protein extracts, immunoblotting with patient serum, and identifying signal-positive bands via mass spectrometry, which require significant time and resource consumption for large-scale studies, given the vast diversity of consumable fish around the world [13,14].

Next-generation sequencing (NGS) methods, particularly RNA sequencing (RNA-seq), show promise in allergen discovery and profiling. In 2020, a study using RNA-seq for five shrimp species identified all seven known crustacean allergens and over 30 potential novel allergens [15]. This approach involves the de novo assembly of transcripts and alignment with known allergens, predicting their allergenicity based on sequence similarity. Compared to classical protein-based methods, the RNA-seq method can reveal comprehensive allergen expression profiles without extraction bias in a more cost-effective way for parallel studies.

In this study, we used RNA-seq to characterize allergen expression profiles of eight widely consumed fish species. Combining clinical ImmunoCAP sIgE data, we explored the association between fish allergen profiles and serological sIgE sensitization patterns, offering insights into rational CRD design and food management for fish allergy.

## 2. Results

### 2.1. Overview of Potential Allergen Transcripts Identified by Fish RNA-Seq Analysis

**Figure 1:**
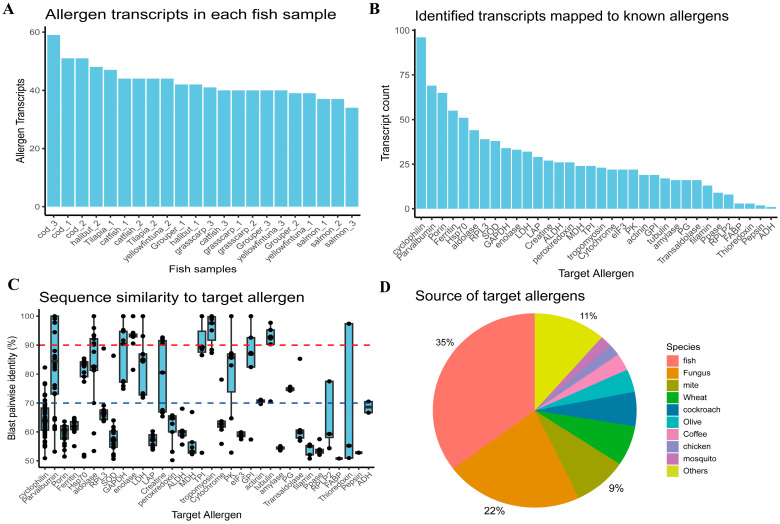
Characterization of identified allergen transcripts across eight fish species. (**A**) Number of putative allergen transcripts identified in each fish sample, ranked in descending order. (**B**) Number of transcripts mapped to different allergen molecules, ranked in descending order. (**C**) Pairwise sequence identities of transcripts mapping to the corresponding reference allergens. The dotted lines represent thresholds of 70% and 90% sequence identity, respectively. (**D**) Pie chart illustrating the top 10 source organisms of targeted allergens according to records in the allergen database. Species are listed in descending order of allergen counts. “Others” refers to the collective category of other species with low counts.

RNA-seq data of eight fish species—yellowfin tuna, salmon, halibut, cod, grouper, grass carp, catfish, and tilapia—were obtained from public datasets [16,17,18,19], each comprising 2–3 samples of fish muscles or whole bodies [Table S1]. Fish transcripts were de novo assembled and aligned against known allergens in the AllergenOnline database [20], following a pipeline similar to that of the previous shrimp allergy study [15]. On average, around 40 potential allergen transcripts were identified per fish species [Figure 1A], mapped to 34 known allergens at the molecular level [Figure 1B]. Cyclophilin had the highest number of transcripts identified, followed by PV, porin, and ferritin, etc. These transcripts exhibited varying sequence identities to their target allergens [Figure 1C]. Cyclophilin transcripts, despite being numerous, had less than 70% identity to their target allergens, indicating lower probabilities of being truly allergenic. Generally, proteins with more than 70% identity to known allergens were considered medium to high probability allergens [20,21,22], including PV, heat shock protein 70 (Hsp70), aldolase, glyceraldehyde-3-phosphate dehydrogenase (GAPDH), enolase, L-lactate dehydrogenase (LDH), creatine, triosephosphate isomerase (TPI), tropomyosin, pyruvate kinase (PK), glucose-6-phosphate isomerase (GPI), alpha-actinin, tubulin, and glycogen phosphorylase-like protein (PG). These putative allergens offered possibilities for CRD design and better characterization of allergen sensitization at the molecular level. In total, approximately 35% of known target allergens were originally identified in fish, with others from distant fungi (22%) and mites (9%), indicating successful fish allergen identification and the possibility to explore the cross-reactivity of different allergen resources [Figure 1D].

To assess the comprehensiveness of transcriptomic allergen profiles, we compared the putative salmon allergen transcripts with the known salmon allergens in the database [Table S2]. Six of the seven recorded salmon allergens were successfully identified, except for the Sal s 6 collagen [23], likely due to its repetitive GXY sequences confusing de novo assembly algorithms [24]. Despite the loss of collagens in all fish, additional 14–28 potential allergens were identified, still revealing informative allergen profiles for addressing allergenic differences.

### 2.2. Overall Fish Allergen Profiles and Top Expressed Allergens Distinguish Fish into Two Categories

**Figure 2:**
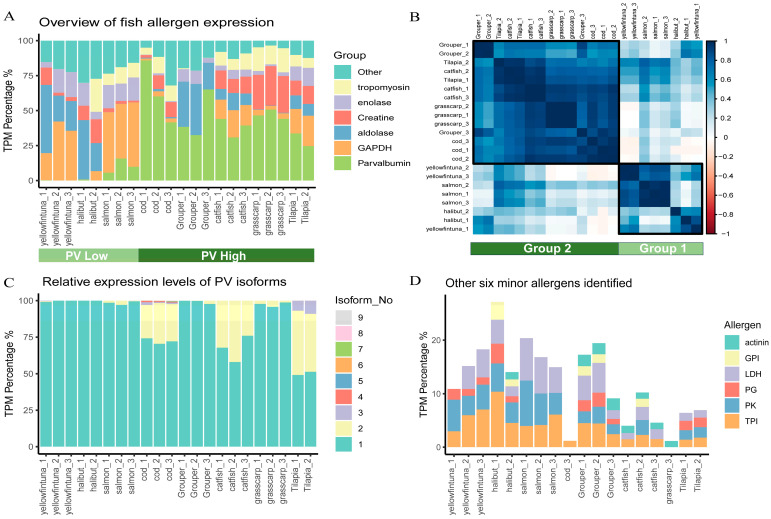
Abundance and profiles of allergen expression. (**A**) Stacked bar plot depicting the relative expression levels of the six highly expressed allergens in each fish samples. The “Other” group represents the cumulative expression of all remaining allergens. Samples were roughly classified into “PV high” and “PV Low” groups based on PV expression. (**B**) Correlation heatmap of allergen expression profiles among fish samples. Fish samples were clustered into two groups by hierarchical clustering. (**C**) Relative expression levels of PV isoforms in each fish sample. PV isoforms are numbered in descending order of their TPM expression values. (**D**) Relative expression levels of potential minor fish allergens: L-lactate dehydrogenase (LDH), triosephosphate isomerase (TPI), pyruvate kinase (PK), glycogen phosphorylase-like protein (PG), and glucose-6-phosphate isomerase (GPI).

The expression levels of these potential allergens were measured by Transcripts Per Million (TPM) to calculate their relative abundances. The six highly expressed allergens accounted for over 70% of total allergen expression in all fish [Figure 2A]. They were PV, GAPDH, aldolase, enolase, creatine, and tropomyosin, all of which had been previously reported as major or minor fish allergens [25,26,27]. Pearson correlation analysis of allergen expression profiles clustered these fish samples into two distinct groups [Figure 2B], consistent with their different PV expression levels [Figure 2A]. Group 1 (tuna, salmon, halibut) had lower PV expression (“PV-low”) and higher expression of other minor allergens, while Group 2 (cod, grouper, grass carp, tilapia, catfish) had dominant PV expression (“PV-high”). This kind of fish classification based on their allergen profile revealed the molecular basis of different fish allergenicity and offered complementary insights on fish avoidance, regarding the traditional classification based on fish source (sea or fresh water) and meat color (white or red muscle) [28,29].

We further explored the isoform expression profiles of these top expressed allergens. For instance, cod expressed up to nine PV isoforms, while the highest expressed isoform dominated 50–90% of the total PV expression [Figure 2C]. For other allergens, the top one isoform exhibited a similarly dominant expression [Figure S1]. Focusing on these top expressed isoforms may simplify the design of CRD.

Additionally, many identified allergens were clustered together as the “other” group due to their low expression [Figure 1A], indicating a lower likelihood of these allergens triggering allergic reactions. By combining insights regarding sequence similarities and expression levels, we manually filtered transcripts with more than 70% sequence identity to known allergens and TPM, accounting for more than 1% of total allergen expression in at least three fish samples as potential “truly” fish allergens. Beyond the six highly expressed allergens, this strategy additionally identified LDH, TPI, PK, PG, GPI, and alpha-actinin as minor fish allergens [Figure 2D]. Notably, PG, which was recently identified as a shrimp allergen [30], may contribute to fish–shrimp cross-reactivity [31].

### 2.3. Fish Allergen Profiles Improve Interpretation of sIgE Sensitization Patterns

**Figure 3:**
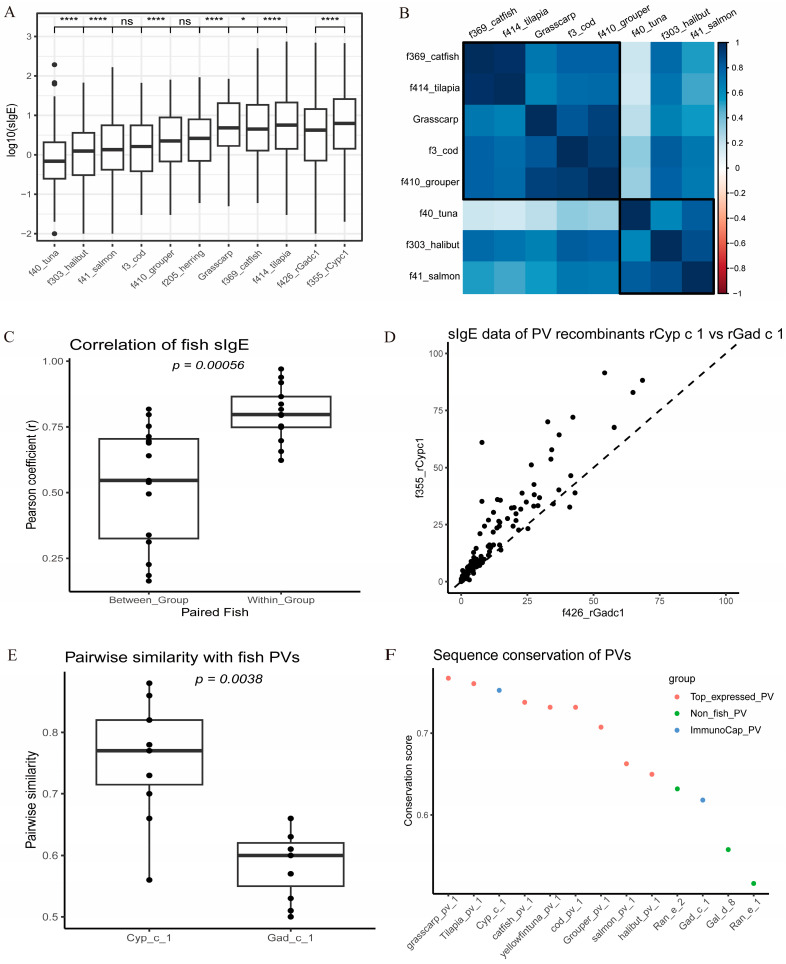
Correlation between fish allergen expression profiles and ImmunoCap sIgE sensitization patterns. (**A**) sIgE gradient of nine fish extracts (ThermoFisher kit ID): tuna (f40), halibut (f303), salmon (f41), cod (f3), grouper (f410), herring (f205), catfish (f369), tilapia (f414), and grass carp (homemade), along with two PV recombinants, rGad c 1 (f426) and rCyp c 1 (f355). Paired comparisons of adjacent groups were marked by Wilcoxon test: * *p* < 0.05, **** *p* < 0.0001, ns: not significant. (**B**) Correlation heatmap of fish extract sIgE data. Fish extracts were clustered into two groups by hierarchical clustering. (**C**) sIgE correlations of paired fish within or between fish groups. Fish groups: tuna, salmon, and halibut (Group 1), and cod, grouper, grass carp, tilapia, and catfish (Group 2). (**D**) Dot plot sIgE data of PV recombinants rCyp c 1 and rGad c 1. The dotted reference line represents a slope of 1. (**E**) Pairwise sequence similarity of Cyp c 1 and Gad c 1 to other fish PVs listed in Table S4. (**F**) Conservation scores of the top one expressed fish PVs (red), Cyp c 1 and Gad c 1 (purple), and frog and chicken PVs (blue).

Routine ImmunoCAP sIgE assays often use multiple fish extracts to address potential cross-reactivity. Our previous study [32] reported a Hong Kong fish allergy cohort tested with nine fish extracts: tuna (f40), halibut (f303), salmon (f41), cod (f3), grouper (f410), herring (f205), catfish (f369), tilapia (f414), and grass carp, along with two PV recombinants: rGad c 1 (f426) and rCyp c 1 (f355). Here, we included sIgE data of 188 subjects who were serologically sensitized (sIgE > 0.35 kUA/L) to at least one fish extract or PV [Figure 3A and Table S3]. We explored whether the fish allergen profile revealed by transcriptomic analysis could help interpret the sIgE sensitization patterns observed.

First, the sIgE levels of tuna, salmon, and halibut extracts were significantly lower than those of grass carp, catfish, and tilapia [Figure 3A]. This serological sIgE gradient was typically attributed to the variable PV content in previous wet-lab studies [33,34], which was also in line with our transcriptomic-based fish classification.

Second, the correlation heatmap of fish sIgE data [Figure 3B] appeared to mimic the correlation figure of allergen expression [Figure 2B], where tuna, salmon, and halibut were clustered together. This observation indicated that paired fish with higher correlations of allergen expression might lead to higher sIgE correlations. Indeed, paired fish within the same fish group (e.g., within Group 1 fish: tuna, salmon, halibut) had significantly higher sIgE correlation coefficients than those of paired fish from different fish groups (e.g., Group 1 fish tuna and Group 2 fish cod) (*p* = 0.00056) [Figure 3C]. The overall association between allergen expression correlation and sIgE correlation was significant (*p* = 0.02), but retained a relatively low coefficient (Pearson r = 0.437) [Figure S2]. Given the variability and potential bias due to fish protein extracts [35], this kind of moderate correlation was considered to be acceptable. The observed differences in fish allergen profiles may help to predict variations in sIgE measurements and reveal distinct fish allergenicity.

Third, the sIgE levels of rCyp c 1 were higher than those of rGad c 1 (*p* < 0.0001) [Figure 3A,D], indicating different binding affinities or sensitization abilities due to differences in PV sequences. A phylogenetic tree of PVs showed that Cyp c 1 is closer to other fish PVs than Gad c 1 [Figure S3A]. Cyp c 1 also had higher pairwise sequence similarities to fish PVs (*p* = 0.0038) [Figure 3E and Table S4]. Probably, the higher sIgE levels of rCyp c 1 resulted from its higher sequence conservation.

To generalize this insight, we constructed the consensus sequence of all PVs in the allergen database [Figure S3B], enabling the calculation of conservation scores for Cyp c 1, Gad c 1, and other PVs, which aimed to quantify the sequence differences of different PVs. The results showed that Cyp c 1 had a higher conservation score than Gad c 1 (0.75 vs. 0.62), while chicken (Gad d 8) and frog PVs (Ran e 1, Ran e 2) had nearly the lowest scores (0.52~0.63) [Figure 3F], in line with the fact that only a few cross-reactivity allergy cases were reported among frog, chicken, and fish [36,37]. The top expressed PV isoforms of grass carp, tilapia, and catfish with higher conservation scores may elicit higher sIgE levels. This prediction matches our previous finding of higher IgE reactivity to grass carp PV (Cten i 1) compared to cod (Gad m 1) and salmon (Sal s 1) [38]. Of note, although tuna PV has a relatively high conservation score (0.73), the expression of tuna PV accounted for less than 1% of total allergen expression [Figure 2A]. Given the low sIgE measurement of tuna extract, both the PV expression and sequence difference shall be taken into consideration for fish allergenicity evaluation.

Overall, the transcriptomic allergen analysis provided comprehensive information about fish allergen expression and allergen sequences for cross-reactivity discussion, advancing the interpretation of clinical sIgE results. Fish with low PV expression and low PV sequence conservation scores, such as salmon and halibut, tended to yield a lower sIgE measurement and may be less allergenic for the fish allergy patients in our cohort, suggesting the potential application of RNA-seq analysis for fish allergenicity prediction and tailored food management.

### 2.4. Diagnosis Performance of sIgE Data Indicated Rational CRD Designs for Different Fish Allergy

Transcriptome analysis revealed certain similarities of allergen expression among different fish. We further explored whether the sIgE data of one representative fish extract was applicable for the allergy diagnosis of other fish, and checked if multiple fish extracts and PVs together could improve sIgE diagnosis performance.

**Figure 4:**
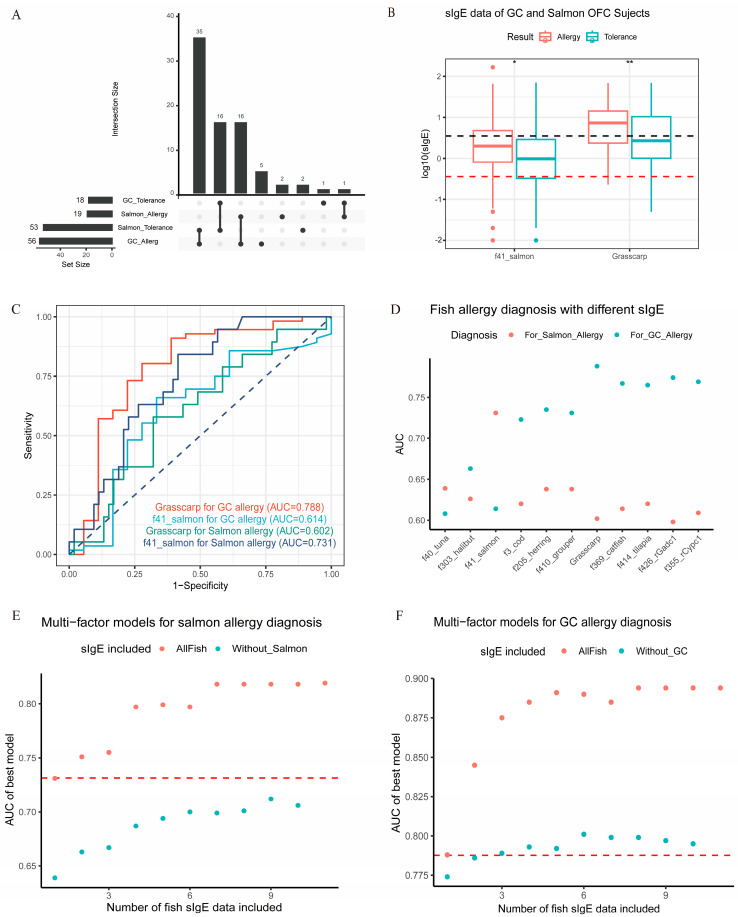
Allergy diagnosis based on multiple sIgE data of fish extracts and PVs. (**A**) Categories of allergy patients based on GC and salmon OFC. (**B**). Boxplot of GC and salmon sIgE data of OFC subjects. Wilcoxon test between allergy and tolerance groups were marked as follows: * *p* < 0.05, ** *p* < 0.01. Dotted lines in red and black colors refer to sIgE levels of 0.35 and 3.5 (kUA/L), respectively. (**C**) ROC curves for allergy diagnosis based on salmon or GC IgE data, AUC values were marked, respectively. Dotted line was provided as reference of AUC value equal to 0.5. (**D**) Performance of different sIgE data for salmon (red) and GC (blue) allergy diagnosis. (**E**) Multi-factor logistic regression models for salmon allergy diagnosis incorporating sIgE data from different fish species, with (red) or without (blue) salmon sIgE included. The red dotted line indicates AUC value of salmon sIgE-only model. (**F**) Multi-factor logistic regression models for GC allergy diagnosis incorporating IgE data from different fish species, with (red) or without (blue) GC sIgE included. The red dotted line indicates the AUC for the GC sIgE-only mode.

In our fish allergy cohort, 78 subjects underwent the grass carp (GC) or salmon oral food challenge (OFC). In addition, 56 of 74 subjects (75.7%) were confirmed as having a GC allergy, and 19 of 72 (26.4%) were confirmed as having a salmon allergy [Figure 4A]. The majority of salmon-allergic patients (16/19) were also allergic to GC, while most GC patients (35/56) were allergic only to GC but not salmon. For corresponding fish sIgE levels, allergic patients received significantly higher values than those of tolerant subjects (*p* < 0.05) [Figure 4B and Table S5].

This OFC-confirmed cohort was selected for Receiver Operating Characteristic (ROC) analysis to evaluate the diagnostic performance of different fish sIgE data by Area Under Curve (AUC) comparison. The results showed that salmon sIgE achieved an AUC of 0.731 for salmon allergy diagnosis, and GC sIgE achieved an AUC of 0.788 for GC allergy diagnosis [Figure 4C]. No other fish or PV sIgE could exhibit a better AUC [Figure 4D].

For GC allergy diagnosis, the sIgE data of two PVs demonstrated similar diagnostic performance (AUC~0.76–0.77) to the GC fish extract. However, for salmon allergy, neither rGad c 1 nor rCyp c 1 sIgE reached comparable performance (AUC < 0.65) to the salmon fish extract. PVs may work as potential alternatives to fish extracts or as CRD candidates for GC and other “PV-high” fish, but not for salmon or “PV-low” fish.

We then applied multi-factor logistic regression models to include all types of sIgE data to explore their best combinations for allergy diagnosis. The results showed that incorporating multiple sIgE data improved diagnostic performance for both salmon and GC allergy, although a maximum AUC ceiling existed even when all sIgE data were included [Figure 4E,F]. For salmon allergy, a combination of other fish sIgE data, excluding salmon data, did not surpass the AUC of salmon sIgE alone [Figure 4E]. For GC allergy diagnosis, using other fish sIgE data achieved an equal or slightly higher AUC than GC sIgE alone [Figure 4F]. These results suggested that sIgE assays based on specific fish extracts remained the best choice for corresponding fish allergy diagnosis. However, sIgE data based on one “representative fish extract” may not be reliable for predictive diagnosis to another fish, especially for “PV-low” fish. Thus, the strategy of CRD, selecting “representative allergens”, seems to be a more promising and complete solution for universal fish allergy diagnosis and prediction.

## 3. Discussion

This study utilized a transcriptomic approach to profile allergen repertoires across eight fish species commonly implicated in fish allergies, which successfully identified and quantified 34 putative allergens, highlighting six highly expressed (PV, GAPDH, aldolase, enolase, creatine, and tropomyosin) and six relatively low-expressed (LDH, TPI, PK, PG, GPI, and alpha-actinin) allergens.

These allergens have demonstrated varying allergy potencies. Serum IgE binding to PVs has been widely reported and characterized in many different fish allergies [39], alongside more clinically relevant support such as SPT and basophil activation tests (BAT) [34], while co-sensitization to collagen, GAPDH, aldolase, enolase, creatine, tropomyosin, LDH, TPI, PK, GPI, or alpha-actinin, etc., was partially described in specific fish allergy studies [25,28,40,41]. Previous studies have reported enolases and aldolases as important fish allergens in cod, salmon, and tuna allergy patients [26]. For 27.4% of patients who had no specific IgE to PVs but did have sIgE to enolases and aldolases, they did not show alleviated reactions but still experienced mild to severe asthmatic symptoms, compared to those of PV-sensitized patients. In addition, it was noteworthy that PV and tropomyosin were also more heat-stable allergens than other allergens such as GAPDH, aldolase, enolase, and creatine [42,43]. The molecular allergenicities can be variable depending on different cooking and consumption scenarios [25,43]. Although PVs are currently regarded as the dominant fish allergen components, the allergen potency of other minor allergens should not be ignored and requires further clarification with more clinical relevance in cohorts with diverse consumption habits.

Except for the loss of collagens, our RNA-seq study offered the first comprehensive allergen profile for multiple fish species under a parallel and comparable framework, providing hints for future validation. Together with feasible access to full-length sequences of these allergens, this high-resolution allergen map provided unlimited possibilities to explore different fish allergenicities in multiple dimensions. The RNA-seq-derived allergen repertoire can serve as a valuable complement to current protein allergen databases established through wet-lab studies. An interesting observation regarding dominantly expressed isoforms helps simplify the candidate selection process for CRD design. For instance, the top expressed isoforms that also share close evolutionary distances with each other may more likely function as pan-allergens across multiple fish species and be useful in clinical practice. Given the transferable features of NGS technology, these results reinforced the potential of the RNA-seq method for allergen mapping among cross-reactive food sources, fish and shellfish for instance, which may share allergens such as tropomyosin, TPI, and PG, etc. [15,44].

Notably, previous studies have shown that fish muscles in different colors (white or red) and different body parts (dorsal, ventral, rostral, or caudal) have variable content of PV proteins [45]. Our current analysis mainly relied on publicly available RNA-seq data of fish muscle or whole-body samples. More comprehensive comparisons of different fish tissues at various fish developmental stages may help reveal more dynamic allergen profiles and potentially identify clinically relevant allergen isoforms in each fish.

Fish can be roughly classified into two groups based on their allergen expression patterns: “PV-high” and “PV-low”. Fish with high PV expression, such as grass carp, tilapia, and catfish, exhibited higher ImmunoCAP sIgE levels in our allergy cohort, in contrast to low PV expression fish, including tuna, salmon, and halibut. A higher correlation of allergen expression was associated with a higher correlation in sIgE values, although the predictive power was moderate. These integrative observations about the allergen profile and sIgE measurement were in line with previous discussion about the “fish allergenicity ladder” [32]. The RNA-seq method potentially provided a cost-effective solution for large-scale fish classification and food management for allergy patients.

As an attempt at fish allergenicity evaluation, we proposed PV consensus sequence and conservation scores to quantify a PV sequence difference. This approach suggested that fish PVs with higher conservation scores might lead to higher sIgE measurements, indicating higher allergenicity or cross-reactivity. Together with the PV expression levels measured by TPMs, these numerical methods may help predict and rank the different fish allergenicities in the future. Of course, the reliability and limitations of proposed PV conservation scores require further tests and clarification. Given the complexity of real-world labeling and processing of fish products, allergic patients should still be reminded to avoid all types of fish in daily life to prevent risky allergic reactions following accidental exposure to their allergic fish. The insight regarding differences in fish allergenicity may benefit better diagnosis and oral immunotherapy for fish allergy in clinical practice.

We also explored whether the sIgE data of one specific fish extract could serve as equivalents or alternatives for other fish allergy diagnoses. Analysis showed that the sIgE data of salmon or GC extract remained the best indicator for diagnosing corresponding fish allergy, however, their predictive power to other fish allergies may be very limited. Moving towards CRD, PV recombinants displayed comparable performance to GC extract for GC allergy diagnosis but performed much worse in salmon allergy diagnosis. This suggests that the rational CRD design should include other minor allergens, as this might be necessary for salmon and “PV-low” fish allergy diagnosis. For instance, selectively incorporating the most highly expressed allergens in salmon, tuna, and halibut—such as aldolase, enolase, and GAPDH—can expand the current CRD panel beyond the two PVs alone. This approach would provide more clinically relevant results that help to clarify the allergenic potential of non-PV allergens as well.

It is important to note that the results regarding sIgE sensitization and OFC diagnosis may only apply to our allergy cohort. Cohorts in other regions with distinct fish consumption habits may exhibit different allergen sensitization patterns [46]. In our cohort, subjects reported the highest frequency of salmon consumption followed by grass carp and grouper, with catfish and tilapia being the least consumed [32]. There is a possibility that individuals initially sensitized to grass carp may have lower reactivity to salmon allergens. The connection between fish consumption habits and differences in intrinsic fish allergenicity remains an open question for further exploration. Moreover, although commercial fish extracts are widely applied for sIgE assays, OFC tests in different cohorts may involve regionally dominant fish species under varying cooking methods [47,48]. For instance, grass carp may be less consumed in European regions while salmon is more popular when served raw, potentially inducing more heat-labile allergens. All these differences should be considered when interpreting the sIgE data and OFC results interactively.

This study has certain limitations and weaknesses related to the use of RNA-seq for allergen discovery. As a sequence alignment method relying on known allergen databases, it is not capable of identifying entirely novel allergens that do not exist in the database or allergens sharing structural epitopes. It may also have limitations in dealing with complex allergen sequences, such as collagen with numerous GXY repeats, due to the short-read nature of current NGS methods. This issue might be better addressed with third-generation long-read sequencing technologies. Compared to traditional wet-lab methods, which may have limitations such as potential bias in protein extraction due to different buffers, relatively low sensitivity due to limited patient serum involvement, and the inability to provide full-length identification with mass spectrometry, the RNA-seq method can serve as a complementary approach. It provides an unbiased allergen profile in large-scale studies, offering full-length allergen sequences and relative abundance at the RNA level, which should benefit the future exploration of possible cross-reactivity among different food sources. Analysis of the sequence details of food and human homologs also offers the opportunity to explore fundamental questions about the molecular basis of allergens with respect to their specificity over other non-allergen molecules. It is worth noting that the association between RNA stability and protein abundance was not discussed in this study. Given the potential variability in protein abundance regarding protein extracts or real-world cooking, the initial state revealed by RNA expression levels may serve as a good reference starting point to explore these effects in various real-world consumption scenarios. Despite these limitations, the RNA-seq approach offers valuable insights into the allergen profiles of various fish species, providing a foundation for further investigations into fish allergenicity and the development of more precise diagnostic tools.

## 4. Materials and Methods

### 4.1. Collection of Fish RNA-Seq and ImmunoCap sIgE Data

The RNA-seq allergen study method offers significant advantages for large-scale profiling. The rapid development and numerous public NGS resources also offer unlimited possibilities to explore allergen differences across various species. In this study, we focus on fish species commonly consumed in Hong Kong, being high in worldwide consumption, and widely studied in sIgE assays to explore their differing allergenicity [49,50]. We obtained RNA-seq data from 8 fish species—yellowfin tuna, halibut, salmon, cod, grouper, catfish, tilapia, and grass carp—from public datasets [16,17,18,19]. For each fish, two or three biological replicates from muscle or whole fish tissue samples were included, labeled as fish names with attributions 1, 2, 3 for further analysis. The RNA-seq data details are summarized in Table S1.

The fish allergy cohort was recruited between 2016 and 2023 from six hospitals in Hong Kong, namely the Prince of Wales Hospital (PWH), Queen Elizabeth Hospital (QEH), Queen Mary Hospital (QMH), Princess Margaret Hospital (PMH), Yan Chai Hospital (YCH), and United Christian Hospital (UCH), as described in our previous report [32]. Subjects with complete ImmunoCAP sIgE data were included, comprising nine fish whole extracts: tuna (f40), halibut (f303), salmon (f41), cod (f3), grouper (f410), herring (f205), catfish (f369), tilapia (f414), and grass carp (for research use only, developed by Thermo Fisher Scientific) [25], along with two recombinant parvalbumins, Gad c 1 (f426) and Cyp c 1 (f355). All sIgE measurements were conducted at the Paediatric Research Laboratory in PWH equipped with the Phadia 200 system. In total, 188 subjects sensitized to at least one fish extract or PV (sIgE > 0.35 kUA/L) were selected for serological sensitization analysis. This sIgE cutoff is widely used in clinical practice to confirm allergic sensitization and report negative results, derived from the initial detection limit of the first sIgE assay [51]. Among them, 74 subjects underwent a grass carp OFC, 72 subjects underwent a salmon OFC, and 68 subjects underwent OFCs for both grass carp and salmon [Figure 4A]. These subjects were included for evaluating the diagnostic performance of different fish sIgE data.

### 4.2. RNA-Seq Data Analysis and Allergen Identification

The RNA-seq data analysis and allergen identification adapted a similar pipeline as the previous study on shrimp allergen discovery [15]. Briefly, the initial quality assessment of raw RNA-seq data employed FastQC, followed by adapter trimming and low-quality read filtering using fastp [52]. Rcorrector then corrected random sequencing errors to ensure data integrity [53]. De novo assembly utilized the Trinity toolkit, and transcript expression was quantified as TPMs using Trinity scripts [54]. TransRate and BUSCO (against the arthropoda odb9 database) evaluated assembly quality [55,56].

A reference allergen database was constructed based on the AllergenOnline databases [20]. The latest version (v22) of the AllergenOnline database contained 2290 allergens identified over the past decades. BLAST searches, with a pairwise identity threshold of 50% and subject coverage exceeding 90%, identified transcripts homologous to known allergens, based on a previous report about the criteria of sequence similarities for allergen prediction [21]. For each transcript and known allergen, only the best BLAST match (with the lowest E-value) was retained to remove duplicate alignments. For allergen isoform analysis, isoforms were numbered in descending order of the expression TPM level. Fish allergen transcript sequences were extracted and translated to amino acid (AA) sequences using the ExPASy translate tool [57] for sequence similarity analysis, multiple alignment, and phylogenetic tree construction with MEGA 14 [58].

### 4.3. Parvalbumin Conservation Score Calculation

In total, 80 unique parvalbumin sequences identified from fish transcriptomes or the AllergenOnline database were included. Multiple sequence alignment was performed using the R msa package and WebLogo to identify the parvalbumin consensus sequence [59,60], in a length of 110 AA. For the AA at each position, the conservation scores were calculated as the AA frequency among all parvalbumins. For a given parvalbumin, the parvalbumin conservation score was calculated as the average AA conservation score across the full length. The conservation scores for chicken (Gad d 8) and frog (Ran e 1 and Ran e 2) parvalbumins were also calculated as a reference for fish parvalbumins.

### 4.4. ROC Analysis

Subjects who underwent grass carp or salmon OFCs were included for sIgE ROC analysis using the R package multipleROC. For multiple sIgE analysis, logistic regression models were applied [61]. The R package bestglm was used to identify the best subset from multiple fish sIgE data under each assigned subset size using the Akaike Information Criterion (AIC) [62]. For comparing the diagnosis performance, the AUC was calculated based on either single fish sIgE data or multiple sIgE logistic regression models accordingly.

### 4.5. Statistics and Figure Plots

Correlation was calculated using the Pearson method in the R package cor. Linear regression models and corresponding parameters were computed using the R package lm. For two-group comparisons, Wilcoxon rank-sum tests were applied using either the single or paired model, as applicable. R packages such as ggplot2 and heatmap were used to generate bar plots, boxplots, dot plots, heatmaps, and other figures presented in this study.

## 5. Conclusions

This study provides comprehensive allergen profiles of eight common fish species using RNA-seq analysis. The high-resolution allergen map, featuring full-length sequences and relative expression abundance at the isoform level, systematically reveals molecular differences in fish allergenicity. This RNA-seq-based allergen repertoire serves as a valuable complement to current protein-based allergen databases and could be further expanded by including more food species in future studies.

The fish allergen profiles facilitated a better interpretation of clinical ImmunoCAP sIgE data, demonstrating potential applications in fish allergenicity prediction and improved fish classification for food avoidance strategies in fish allergy management. Regarding future CRD design for different fish allergies, our analysis suggests that the current PV-only panel may be primarily applicable for PV-high fish species. Including other minor allergens such as aldolase and enolase, etc., could be a promising direction for future CRD development.

These findings highlight the potential of large-scale RNA-seq analysis for fish allergenicity prediction, classification, and food management. Future studies should incorporate more clinical evidence to clarify the allergenic potency of different minor allergens across multiple allergy cohorts and at the protein level if possible. Such research will advance our understanding of the molecular basis of fish allergies and contribute to improved diagnostic and management strategies for fish allergy patients.

## Data Availability

Summary statistics of allergen profiles and sIgE patterns are presented in the main figures and Supplementary Materials. Raw data of fish RNA-seq can be freely downloaded from the GEO database (https://www.ncbi.nlm.nih.gov/geo/, accessed on 1 July 2023) using the accession numbers provided in Table S1. Clinical demographic data, symptoms, sIgE measurements, and OFC outcomes of allergy subjects can be obtained from the authors upon reasonable request, subject to ethical restrictions.

## Supplementary Materials

The following supporting information can be downloaded at: https://www.mdpi.com/article/10.3390/ijms251910784/s1.
