# Supplementary material for: Combined Integrative RNA-Seq and Serological sIgE Analysis Enhances Understanding of Fish Allergen Profiles and Diagnostic Strategy for Fish Allergy
Source: Int J Mol Sci. 2024 Oct 7;25(19):10784. doi: 10.3390/ijms251910784 (PMC11477408; doi:10.3390/ijms251910784)
Supplement: Supplementary file 1 [file ijms-25-10784-s001.zip › ijms-3226563-supplementary.pdf]

Supplementary Tables

Table S1. Summary of RNA-seq data

Fish	Species name	SRA	Sample name	Tissue
Catfish	Pangasianodon hypophthalmus	SRR22306773	catfish_1	muscle
		SRR22306775	catfish_2	muscle
		SRR22306801	catfish_3	muscle
Cod	Gadus morhua	SRR2225010	cod_1	whole body mix
		SRR1913709	cod_2	whole boby mix
		SRR1913821	cod_3	whole body mix
Grasscarp	Ctenopharyngodon idella	SRR17257184	grasscarp_1	muscle
		SRR17257185	grasscarp_2	muscle
		SRR17257182	grasscarp_3	muscle
Grouper	Epinephelus fuscoguttatus	SRR19851487	Grouper_1	white muscle
		SRR19851485	Grouper_2	white muscle
		SRR19851482	Grouper_3	white muscle
Halibut	Hippoglossus stenolepis	SRR11826189	halibut_1	pooled white muscle
		SRR11826183	halibut_2	pooled red muscle
Salmon	Salmo salar	SRR16287800	salmon_1	white muscle
		SRR16287801	salmon_2	red muscle
		SRR16287802	salmon_3	red muscle
Tilapia	Oreochromis mossambicus	DRR164264	Tilapia_1	muscle
		DRR164259	Tilapia_2	muscle
Yellowfintuna	Thunnus albacares	SRR8756174	yellowfintuna_1	white muscle
		SRR8756186	yellowfintuna_2	white muscle
		SRR8756187	yellowfintuna_3	white muscle

Table S2. Identified potential allergen transcripts in the salmon sample.

<table>
<tr><th>Number of hit</th><th>Transcript_id</th><th>Target_allergen_id</th><th>Species</th><th>Allergen_name</th><th>Identity (%)</th><th>Coverage (%)</th><th>Allergen_group</th></tr>
<tr><td>1</td><td>TRINITY_DN40586_c2_g1_i1</td><td>ACI68103.1</td><td>Salmo salar</td><td>Salmo Sal s 1</td><td>100</td><td>100.00</td><td rowspan="2">Parvalbumin</td></tr>
<tr><td>2</td><td>TRINITY_DN50144_c0_g1_i1</td><td>sp|Q91483.3|PRVB2_SALS</td><td>Salmo salar</td><td>Salmo Sal s 1</td><td>100</td><td>100.00</td></tr>
<tr><td>3</td><td>TRINITY_DN53410_c16_g1_i1</td><td>ACH70931.1</td><td>Salmo salar</td><td>Salmo Sal s 2 enolase</td><td>100</td><td>100.00</td><td>enolase</td></tr>
<tr><td>4</td><td>TRINITY_DN123396_c26_g1_i1</td><td>NP_001117128.1</td><td>Salmo salar</td><td>Sal s 4</td><td>100</td><td>96.48</td><td>tropomyosin</td></tr>
<tr><td>5</td><td>TRINITY_DN50678_c45_g1_i1</td><td>XP_026782131.1</td><td>Pangasianodon hypop</td><td>Pan h 13</td><td>91.62</td><td>100.30</td><td>GAPDH</td></tr>
<tr><td>7</td><td>TRINITY_DN55711_c0_g1_i1</td><td>XP_026771637.1</td><td>Pangasianodon hypop</td><td>Pan h 3</td><td>92.18</td><td>80.77</td><td>aldolase</td></tr>
<tr><td>9</td><td>TRINITY_DN59754_c44_g4_i1</td><td>XP_026780620.1</td><td>Pangasianodon hypop</td><td>Pan h 7</td><td>65.4</td><td>96.58</td><td>Creatine</td></tr>
<tr><td>6</td><td>TRINITY_DN58315_c0_g1_i1</td><td>AOD75396.1</td><td>Tyrophagus putrescen</td><td>Tyrophagus putrescentiae Tyr p 35</td><td>58.46</td><td>98.56</td><td>ALDH</td></tr>
<tr><td>8</td><td>TRINITY_DN46442_c0_g1_i1</td><td>ABC68516.1</td><td>Blattella germanica</td><td>Blattella Bla g 11 alpha Amylase</td><td>54.65</td><td>98.06</td><td>amylase</td></tr>
<tr><td>10</td><td>TRINITY_DN49091_c0_g2_i1</td><td>AEY79726.1</td><td>Daucus carota</td><td>Daucus cyclophilin</td><td>72.94</td><td>99.42</td><td rowspan="7">cyclophilin</td></tr>
<tr><td>11</td><td>TRINITY_DN50231_c0_g1_i1</td><td>UZC36341.1</td><td>Periplaneta american</td><td>Per a 18.01 Cyclophilin</td><td>69.52</td><td>90.78</td></tr>
<tr><td>12</td><td>TRINITY_DN48702_c0_g1_i1</td><td>AAP35065.1</td><td>Dermatophagoides fa</td><td>Dermatophagoides Der f 29</td><td>66.25</td><td>97.56</td></tr>
<tr><td>13</td><td>TRINITY_DN59040_c0_g1_i1</td><td>AVV30163.1</td><td>Olea europaea</td><td>Olea Ole e 15 cyclophilin</td><td>65.09</td><td>98.26</td></tr>
<tr><td>14</td><td>TRINITY_DN59088_c0_g4_i1</td><td>AAP35065.1</td><td>Dermatophagoides fa</td><td>Dermatophagoides Der f 29</td><td>63.19</td><td>99.39</td></tr>
<tr><td>15</td><td>TRINITY_DN34826_c0_g1_i1</td><td>AAP35065.1</td><td>Dermatophagoides fa</td><td>Dermatophagoides Der f 29</td><td>62.11</td><td>98.17</td></tr>
<tr><td>16</td><td>TRINITY_DN49904_c0_g3_i1</td><td>AVV30163.1</td><td>Olea europaea</td><td>Olea Ole e 15 cyclophilin</td><td>61.18</td><td>98.84</td></tr>
<tr><td>17</td><td>TRINITY_DN52387_c3_g1_i1</td><td>AAK67492.1</td><td>Cochliobolus lunatus</td><td>Curvularia lunatua Cur l 3 Cochliobolus</td><td>61.76</td><td>94.44</td><td>Cytochrome</td></tr>
<tr><td>18</td><td>TRINITY_DN6626_c0_g1_i1</td><td>ACD65081.1</td><td>Forcipomyia taiwana</td><td>Forcipomyia For t 2</td><td>57.45</td><td>99.08</td><td>eIF3</td></tr>
<tr><td>19</td><td>TRINITY_DN48568_c0_g1_i1</td><td>AAG02250.1</td><td>Dermatophagoides pt</td><td>Der f 30</td><td>64.16</td><td>96.11</td><td>Ferritin</td></tr>
<tr><td>20</td><td>TRINITY_DN56712_c1_g3_i1</td><td>QFI57017.1</td><td>Scylla paramamosain</td><td>Scylla paramamosain filamin C</td><td>55.07</td><td>100.00</td><td>filamin</td></tr>
<tr><td>21</td><td>TRINITY_DN52764_c2_g1_i1</td><td>AOD75395.1</td><td>Tyrophagus putrescen</td><td>Tyrophagus putrescentiae Tyr p 28</td><td>84.26</td><td>92.56</td><td>Hsp70</td></tr>
<tr><td>22</td><td>TRINITY_DN58114_c0_g1_i1</td><td>XP_001657556.2</td><td>Aedes aegypti</td><td>Aedes Aed a 11 Lysosomal protease</td><td>58.99</td><td>97.67</td><td>LAP</td></tr>
<tr><td>23</td><td>TRINITY_DN50407_c0_g1_i1</td><td>XP_001657556.2</td><td>Aedes aegypti</td><td>Aedes Aed a 11 Lysosomal protease</td><td>55.32</td><td>97.16</td><td>LAP</td></tr>
<tr><td>24</td><td>TRINITY_DN51794_c3_g1_i1</td><td>XP_026774991.1</td><td>Pangasianodon hypop</td><td>Pangasianodon Pan h 10</td><td>84.36</td><td>97.90</td><td rowspan="2">LDH</td></tr>
<tr><td>25</td><td>TRINITY_DN56977_c1_g1_i1</td><td>XP_026774991.1</td><td>Pangasianodon hypop</td><td>Pangasianodon Pan h 10</td><td>72.81</td><td>99.40</td></tr>
<tr><td>26</td><td>TRINITY_DN53886_c0_g1_i4</td><td>AAD25927.1</td><td>Malassezia furfur</td><td>Malassezia Mala f 4</td><td>52.34</td><td>100.00</td><td>MDH</td></tr>
<tr><td>27</td><td>TRINITY_DN20435_c0_g1_i1</td><td>UZC36343.1</td><td>Periplaneta american</td><td>Per a 20.01 Peroxiredoxin</td><td>62.84</td><td>99.09</td><td>peroxiredoxin</td></tr>
<tr><td>28</td><td>TRINITY_DN5553_c2_g1_i1</td><td>XP_026775867.1</td><td>Pangasianodon hypop</td><td>Pan h 9</td><td>85.66</td><td>100.00</td><td>PK</td></tr>
<tr><td>29</td><td>TRINITY_DN55182_c0_g1_i1</td><td>UZC36342.1</td><td>Periplaneta american</td><td>Per a 19.01 Porin 3</td><td>60.61</td><td>97.06</td><td rowspan="3">Porin</td></tr>
<tr><td>30</td><td>TRINITY_DN58600_c1_g1_i1</td><td>UZC36342.1</td><td>Periplaneta american</td><td>Per a 19.01 Porin 3</td><td>58.21</td><td>98.53</td></tr>
<tr><td>31</td><td>TRINITY_DN49750_c0_g1_i1</td><td>UZC36342.1</td><td>Periplaneta american</td><td>Per a 19.01 Porin 3</td><td>56.06</td><td>97.06</td></tr>
<tr><td>32</td><td>TRINITY_DN55813_c0_g1_i1</td><td>AIO08849.1</td><td>Dermatophagoides fa</td><td>Der f 32</td><td>52.1</td><td>100.00</td><td>Ppase</td></tr>
<tr><td>33</td><td>TRINITY_DN45994_c0_g1_i1</td><td>sp|Q8NKF4.2|RL3_ASPFU</td><td>Aspergillus fumigatus</td><td>Aspergillus Asp f 23</td><td>64.39</td><td>100.00</td><td>RPL3</td></tr>
<tr><td>34</td><td>TRINITY_DN40523_c0_g2_i1</td><td>QVG59417.1</td><td>Ambrosia trifida</td><td>Acacia (Vachellia) profilin Aca f 2</td><td>60.67</td><td>98.04</td><td rowspan="2">SOD</td></tr>
<tr><td>35</td><td>TRINITY_DN59751_c0_g1_i1</td><td>ABR29644.1</td><td>Pistacia vera</td><td>Pistacia Pis v 4</td><td>57.83</td><td>100.00</td></tr>
<tr><td>36</td><td>TRINITY_DN40142_c0_g1_i1</td><td>CBW45298.1</td><td>Plodia interpunctella</td><td>Plodia Plo i 2 thioredoxin</td><td>50.98</td><td>96.23</td><td>Thioredoxin</td></tr>
<tr><td>37</td><td>TRINITY_DN55491_c17_g1_i1</td><td>ACM09737.1</td><td>Salmo salar</td><td>Salmon Sal s 8 Triosphosphate isomerase</td><td>100</td><td>100.00</td><td>TPI</td></tr>
</table>

-Allergens highlighted in red refer to reported salmon allergens

Table S3. Summary of ImmunoCap sIgE data of 188 sensitization subjects and 78 oral food challenge subjects based on fish extract and parvalbumin recombinants

name	levels	Sensitized_subjects (N=188)	OFC_subjects (N=78)	p
f3_cod	Median (IQR)	1.6 (0.4 to 5.6)	1.5 (0.4 to 4.7)	.629
f40_tuna	Median (IQR)	0.7 (0.2 to 2.1)	0.6 (0.2 to 1.5)	.432
f41_salmon	Median (IQR)	1.3 (0.4 to 5.6)	1.4 (0.5 to 4.6)	.763
f414_tilapia	Median (IQR)	5.7 (1.4 to 21.6)	5.1 (1.5 to 13.7)	.436
f355_rCypc1	Median (IQR)	6.2 (1.4 to 26.2)	5.1 (1.7 to 14.6)	.364
f426_rGadc1	Median (IQR)	4.2 (0.7 to 14.4)	3.5 (1.0 to 10.2)	.369
Grasscarp	Median (IQR)	4.8 (1.7 to 20.6)	4.4 (1.5 to 11.9)	.378
f303_halibut	Median (IQR)	1.2 (0.3 to 3.6)	0.9 (0.3 to 2.7)	.393
f205_herring	Median (IQR)	2.6 (0.7 to 8.0)	2.7 (0.7 to 5.6)	.573
f410_grouper	Median (IQR)	2.2 (0.6 to 9.0)	2.3 (0.8 to 6.2)	.622
f369_catfish	Median (IQR)	4.5 (1.3 to 18.8)	4.2 (1.3 to 10.4)	.442

-Unit of sIgE data in kUA/L

Table S4. Sequence similarity of fish parvalbumins, ImmunoCap parvalbumins Cyp c 1 and Gad c 1, and frog and chicken parvalbumins

PV_type	PV_source	PV_name	PV_no	1	2	3	4	5	6	7	8	9	10	11	12	13	14	15	16	17	18	19
Transciptome identified Fish PV	Tilapia	Tilapia_pv_1	1	1.00	0.75	0.76	0.80	0.56	0.81	0.85	0.74	0.71	0.69	0.77	0.86	0.88	0.66	0.81	0.69	0.53	0.50	0.68
		Tilapia_pv_2	2	0.75	1.00	0.73	0.80	0.55	0.74	0.76	0.83	0.63	0.65	0.84	0.75	0.73	0.57	0.74	0.65	0.54	0.50	0.62
		Tilapia_pv_3	3	0.76	0.73	1.00	0.75	0.59	0.72	0.78	0.78	0.77	0.69	0.75	0.77	0.82	0.61	0.72	0.69	0.58	0.49	0.61
	Catfish	catfish_pv_1	4	0.80	0.80	0.75	1.00	0.57	0.77	0.88	0.81	0.65	0.66	0.81	0.87	0.78	0.63	0.77	0.66	0.55	0.49	0.64
		catfish_pv_2	5	0.56	0.55	0.59	0.57	1.00	0.49	0.57	0.55	0.52	0.57	0.56	0.57	0.56	0.50	0.49	0.57	0.66	0.58	0.50
	Cod	cod_pv_1	6	0.81	0.74	0.72	0.77	0.49	1.00	0.81	0.72	0.67	0.65	0.75	0.82	0.82	0.61	1.00	0.65	0.53	0.51	0.62
	Grasscarp	grasscarp_pv_1	7	0.85	0.76	0.78	0.88	0.57	0.81	1.00	0.80	0.69	0.70	0.82	0.99	0.86	0.66	0.81	0.70	0.57	0.50	0.68
	Grouper	Grouper_pv_1	8	0.74	0.83	0.78	0.81	0.55	0.72	0.80	1.00	0.68	0.67	0.86	0.79	0.73	0.57	0.72	0.67	0.53	0.45	0.60
	Halibut	halibut_pv_1	9	0.71	0.63	0.77	0.65	0.52	0.67	0.69	0.68	1.00	0.63	0.65	0.69	0.70	0.53	0.67	0.63	0.54	0.52	0.58
	Salmon	salmon_pv_1	10	0.69	0.65	0.69	0.66	0.57	0.65	0.70	0.67	0.63	1.00	0.69	0.70	0.66	0.51	0.65	1.00	0.53	0.50	0.59
	Tuna	yellowfintuna_pv_1	11	0.77	0.84	0.75	0.81	0.56	0.75	0.82	0.86	0.65	0.69	1.00	0.82	0.77	0.60	0.75	0.69	0.55	0.51	0.62
Reference PV from fish in database	Grass carp	Cten_i_1	12	0.86	0.75	0.77	0.87	0.57	0.82	0.99	0.79	0.69	0.70	0.82	1.00	0.87	0.67	0.82	0.70	0.56	0.50	0.69
	Common carp	Cyp_c_1	13	0.88	0.73	0.82	0.78	0.56	0.82	0.86	0.73	0.70	0.66	0.77	0.87	1.00	0.65	0.82	0.66	0.54	0.48	0.66
	Baltic cod	Gad_c_1	14	0.66	0.57	0.61	0.63	0.50	0.61	0.66	0.57	0.53	0.51	0.60	0.67	0.65	1.00	0.61	0.51	0.48	0.42	0.55
	Atlantic cod	Gad_m_1	15	0.81	0.74	0.72	0.77	0.49	1.00	0.81	0.72	0.67	0.65	0.75	0.82	0.82	0.61	1.00	0.65	0.53	0.51	0.62
	Atlantic salmon	Sal_s_1	16	0.69	0.65	0.69	0.66	0.57	0.65	0.70	0.67	0.63	1.00	0.69	0.70	0.66	0.51	0.65	1.00	0.53	0.50	0.59
Reference PV from non-fish in database	Chicken PV	Gal_d_8	17	0.53	0.54	0.58	0.55	0.66	0.53	0.57	0.53	0.54	0.53	0.55	0.56	0.54	0.48	0.53	0.53	1.00	0.73	0.55
	Frog PV	Ran_e_1	18	0.50	0.50	0.49	0.49	0.58	0.51	0.50	0.45	0.52	0.50	0.51	0.50	0.48	0.42	0.51	0.50	0.73	1.00	0.51
		Ran_e_2	19	0.68	0.62	0.61	0.64	0.50	0.62	0.68	0.60	0.58	0.59	0.62	0.69	0.66	0.55	0.62	0.59	0.55	0.51	1.00

- Isoforms of Fish PVs were named by fish name with numbers to indicate their rank of relative expression levels.

Table S5. Summary of ImmunoCap sIgE data of oral food challenge subjects based on fish extract and parvalbumin recombinants

A. Grass carp OFC subjects

name	levels	A (N=56)	T (N=18)	total (N=74)	p
f3_cod	Median (IQR)	2.1 (0.7 to 4.9)	0.4 (0.1 to 1.5)	1.9 (0.4 to 8.9)	.005
f40_tuna	Median (IQR)	0.6 (0.3 to 1.5)	0.3 (0.1 to 0.9)	0.8 (0.2 to 2.8)	.172
f41_salmon	Median (IQR)	1.7 (0.6 to 4.3)	0.7 (0.1 to 1.7)	1.5 (0.4 to 8.1)	.149
f414_tilapia	Median (IQR)	6.9 (2.1 to 14.3)	1.1 (0.8 to 2.6)	5.9 (1.4 to 32.6)	<.001
f355_rCypc1	Median (IQR)	6.8 (2.2 to 15.2)	1.1 (0.7 to 3.4)	6.6 (1.3 to 35.2)	<.001
f426_rGadc1	Median (IQR)	4.2 (1.6 to 10.3)	0.6 (0.2 to 2.3)	4.7 (0.6 to 25.2)	<.001
Grasscarp	Median (IQR)	5.9 (2.0 to 12.8)	1.1 (0.6 to 2.1)	5.3 (1.7 to 23.4)	<.001
f303_halibut	Median (IQR)	1.2 (0.5 to 2.8)	0.3 (0.2 to 0.9)	1.5 (0.3 to 6.0)	.039
f205_herring	Median (IQR)	3.3 (1.0 to 5.8)	0.6 (0.3 to 1.8)	2.7 (0.6 to 11.1)	.003
f410_grouper	Median (IQR)	3.0 (1.3 to 6.4)	0.6 (0.3 to 2.0)	2.4 (0.6 to 12.8)	.003
f369_catfish	Median (IQR)	5.2 (2.2 to 12.0)	0.8 (0.6 to 2.5)	4.9 (1.3 to 26.6)	<.001

B. Salmon OFC subjects

name	levels	A (N=19)	T (N=53)	total (N=72)	p
f3_cod	Median (IQR)	3.5 (1.2 to 5.3)	1.4 (0.5 to 3.1)	1.7 (0.3 to 7.7)	.124
f40_tuna	Median (IQR)	1.0 (0.6 to 2.5)	0.6 (0.2 to 1.3)	0.7 (0.2 to 2.5)	.075
f41_salmon	Median (IQR)	4.5 (1.8 to 6.7)	1.1 (0.5 to 3.4)	1.2 (0.3 to 6.8)	.003
f414_tilapia	Median (IQR)	10.7 (3.3 to 19.2)	4.6 (1.5 to 10.9)	5.5 (1.3 to 31.9)	.125
f355_rCypc1	Median (IQR)	9.8 (4.5 to 18.9)	4.9 (1.8 to 9.8)	6.4 (1.1 to 34.5)	.160
f426_rGadc1	Median (IQR)	5.4 (2.9 to 11.2)	2.9 (1.0 to 7.2)	4.5 (0.6 to 23.8)	.211
Grasscarp	Median (IQR)	9.0 (2.8 to 18.0)	4.3 (1.7 to 11.1)	4.9 (1.4 to 22.8)	.192
f303_halibut	Median (IQR)	1.9 (0.9 to 3.0)	0.7 (0.3 to 2.4)	1.3 (0.2 to 4.9)	.107
f205_herring	Median (IQR)	4.6 (1.8 to 6.6)	2.6 (0.7 to 5.3)	2.4 (0.4 to 10.9)	.077
f410_grouper	Median (IQR)	5.1 (1.8 to 8.9)	2.0 (0.8 to 5.8)	2.1 (0.5 to 11.6)	.077
f369_catfish	Median (IQR)	9.2 (3.2 to 14.7)	4.2 (1.3 to 9.4)	4.6 (1.1 to 24.5)	.143

- Unit of sIgE data: kUA/L

Supplementary Figures

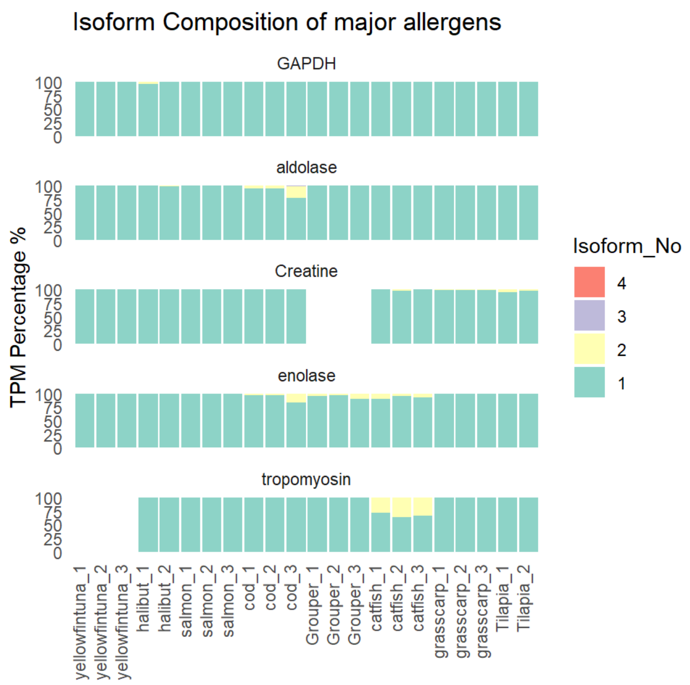

Figure S1. Isoform composition of the highly expressed allergens.

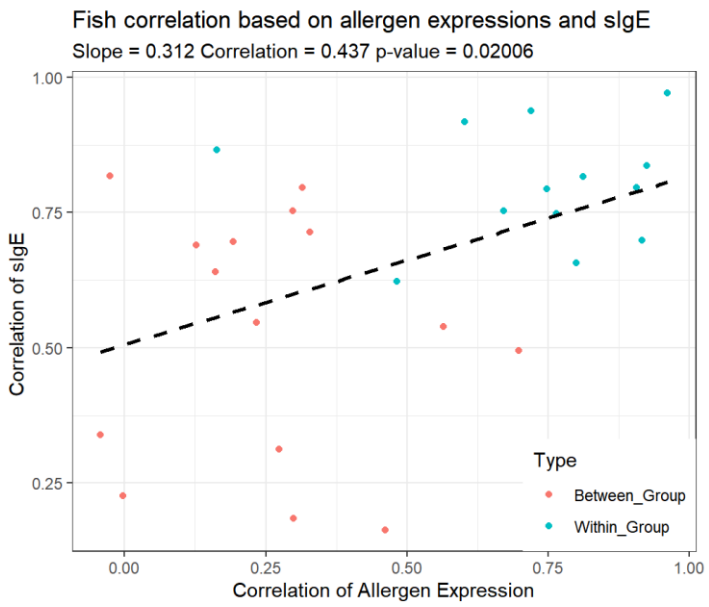

Figure S2. Association between fish allergen expression correlation and fish sIgE correlation for different fish pairs. Fish groups: tuna, salmon, and halibut (Group 1), and cod, grouper, grass carp, tilapia, and catfish (Group 2).

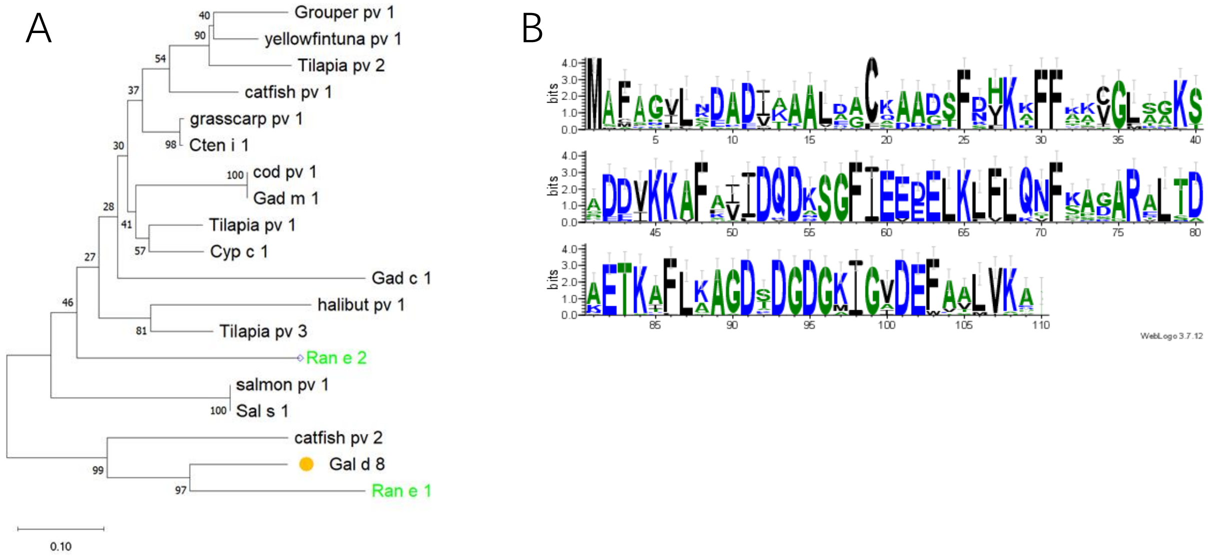

Figure S3. (A) Phylogenetic tree and (B) consensus sequence of parvalbumins.
